# Vitamin A Derivatives and Adipose Tissue Differentiation: Molecular Pathways Driving Browning and Anti-Obesity Effects

**DOI:** 10.1007/s13679-025-00684-2

**Published:** 2026-01-15

**Authors:** Billur Bilikozen Aygun, Berrak Basturk, Aylin Ayaz

**Affiliations:** 1 Department of Nutrition and Dietetics, Institute of of Health Sciences, Hacettepe University, Ankara, Türkiye; 2 Department of Nutrition and Dietetics, Faculty of Health Sciences, Halic University, Istanbul, Türkiye; 3 Department of Nutrition and Dietetics, Faculty of Health Sciences, Hacettepe University, Ankara, Türkiye

**Keywords:** Vitamin A, Adiposity, Carotenoids, Obesity, Browning, Thermogenesis

## Abstract

**Purpose of the Review:**

This review aims to evaluate the effects of retinoids and carotenoids (β-carotene, lycopene, lutein, and β-cryptoxanthin) on adipose tissue biology, particularly browning processes, in light of the current literature. In the current era of increasing global obesity prevalence, understanding the potential regulatory roles of these lipophilic micronutrients in energy homeostasis and adipose tissue plasticity may contribute to the development of new nutrition-based or pharmacological strategies.

**Recent Findings:**

The regulatory roles of vitamin A (VA) and carotenoids in adipose tissue metabolism have been intensively investigated in recent years. Current findings indicate that compounds such as retinoic acid, lycopene, β-cryptoxanthin, and zeaxanthin reduce adipogenesis and lipogenesis by suppressing the expression of peroxisome proliferator-activated receptor gamma (PPARγ), CCAAT/enhancer binding protein alpha (C/EBPα), and sterol regulatory element-binding protein 1c (SREBP1-c) while increasing energy expenditure and promoting the browning of white adipose tissue through the activation of 5′-adenosine monophosphate-activated protein kinase (AMPK) and the upregulation of thermogenic genes such as uncoupling protein 1 (UCP1), PR domain containing 16 (PRDM16), and PPARγ coactivator-1α (PGC-1α). These effects are associated with reduced adipocyte hypertrophy, increased mitochondrial activity, and decreased systemic inflammation.

**Summary:**

VA and carotenoids exert multifaceted effects on adipose tissue differentiation and energy metabolism, suggesting an alternative pathway against obesity. By reducing lipid storage, stimulating thermogenesis, and enhancing oxidative metabolism, these bioactive compounds help restore metabolic balance. Overall, evidence indicates that VA derivatives and carotenoids, through adipose tissue browning and improved metabolic efficiency, represent promising nutrition-based strategies to combat obesity.

## Introduction

Obesity is a condition characterized by abnormal or excessive white fat accumulation or abnormal fat distribution in the body, significantly increasing the risk of developing metabolic diseases [1]. The World Obesity Federation defines obesity as a chronic, relapsing disease process [2]. According to World Health Organization statistics, the global prevalence of obesity more than doubled between 1990 and 2022 [3]. According to the 2022 Turkey Health Survey, the proportion of obese individuals was 21.1% in 2019 and 20.2% in 2022 [4]. The World Obesity Atlas 2025 projects that 36% of adults in Turkey are currently living with obesity, and approximately 24.8 million people are expected to be obese by 2030. Globally, this number is predicted to reach 1.1 billion adults [5]. The increasing prevalence of obesity poses a serious public health threat [6]. It is also a risk factor for many noncommunicable comorbidities, including cancers, ischemic heart disease, stroke, type 2 diabetes, musculoskeletal disorders, and liver and kidney diseases. Combating obesity is a critical step toward reducing the global burden of chronic diseases [5].

Obesity is a multifactorial disorder that arises from the interaction of numerous factors, including genetic predisposition and lifestyle components. Therefore, its treatment and prevention are complex processes [7]. Current therapeutic options include behavioral therapy, lifestyle counseling, pharmacotherapy for body weight management, and metabolic surgery [8]. However, one of the key elements in obesity management is understanding the structural and functional characteristics of adipose tissue. This is because adipose tissue is not merely a passive storage site from which excess energy is to be eliminated but rather a dynamic system that provides an evolutionary advantage by maintaining energy homeostasis and thermoregulation.

In the pathophysiology of obesity, white adipose tissue (WAT) undergoes a remodeling process in response to excessive energy intake and lipid accumulation. This remodeling is directly associated with obesity and occurs through hypertrophy (increase in adipocyte size), hyperplasia (increase in adipocyte number), or a combination of both mechanisms [9, 10]. WAT is not merely an energy reservoir; it also functions as a regulator of energy homeostasis, a potent endocrine organ, and an integral component of many physiological processes [6]. In contrast, brown adipose tissue (BAT) is more metabolically active because of its high mitochondrial density and UCP1-mediated thermogenesis. The rediscovery of BAT in adult humans has increased interest in the transdifferentiation potential of WAT through the browning process. This process has emerged as a novel and promising target for the treatment of obesity and related metabolic disorders [11, 12]. Furthermore, numerous pharmacological agents and nutrition-related compounds capable of promoting browning are being investigated in both clinical and experimental studies [13].

This study aimed to comprehensively examine the effects of VA and its derivatives on BAT activation and the browning process of WAT. In this context, both in vivo and in vitro experimental models have been utilized to evaluate the contributions of VA and its derivatives to adipose tissue metabolism, to investigate the role of brown fat in energy expenditure and thermogenesis, and to explore the trans differentiation potential of WAT. The findings obtained are intended to highlight the potential therapeutic value of VA derivatives in the treatment of obesity and related metabolic disorders.

## Methods

The review was conducted to evaluate the effects of VA and carotenoids (β-carotene, lycopene, lutein, and β-cryptoxanthin) on adipose tissue biology, particularly on the browning and thermogenesis processes of white adipose tissue. Articles published between 2015 and 2025 were searched in the PubMed, Web of Science and Google Scholar databases. The following keywords were used in the search: “vitamin a,” “adiposity,” “carotenoids,” “obesity,” “browning,” and “thermogenesis.” Experimental studies (cell culture, animal models, and a small number of clinical trials) published in English were included. Titles and abstracts were assessed for relevance to the topic, and eligible full-text studies were examined in detail. A large number of studies in the literature were reviewed, and after a comprehensive review, a total of 84 references were used in the study. Among these, 20 basic experimental and clinical studies are summarized in more detail in Table 1, in terms of content, methods, and findings. During data extraction, the study model (in vitro, in vivo, or clinical), intervention type and dose, duration, and findings related to energy metabolism, lipid accumulation, mitochondrial activity, thermogenic gene expression (UCP1, PGC-1α, PRDM16, AMPK), and adipose tissue browning were taken into account.

**Table 1.**
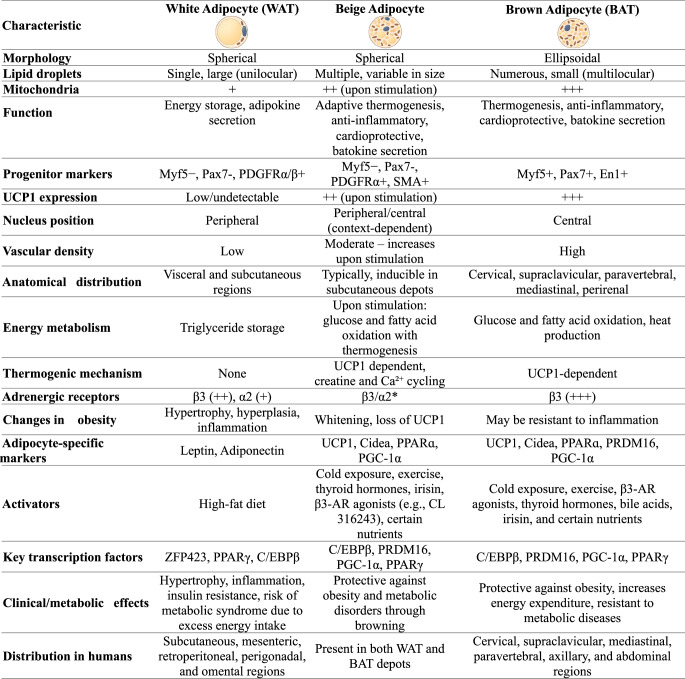
Morphological and functional characteristics of human adipocyte types [17, 18]

*Preliminary findings Relative amounts: + (low), ++ (moderate),+++ (high) Abbreviations: BAT: Brown adipose tissue, C/EBPβ: CCAAT/enhancer-binding protein beta, Cidea: Cell death–inducing DNA fragmentation factor alpha-like effector A, En1: Engrailed-1, Myf5: Myogenic factor 5, Pax7: Paired box protein 7, PDGFRα/β: Platelet-derived growth factor receptor alpha/beta, PGC-1α: Peroxisome proliferator–activated receptor gamma coactivator 1-alpha, PPARɑ: Peroxisome proliferator–activated receptor alfa, PRDM16: PR domain–containing protein 16, SMA: Smooth muscle actin, UCP1: Uncoupling protein 1, WAT: White adipose tissue, ZFP423: Zinc finger protein 423, β3-AR: Beta-3 adrenergic receptor

### Overview of Adipose Tissue

**Fig. 1:**
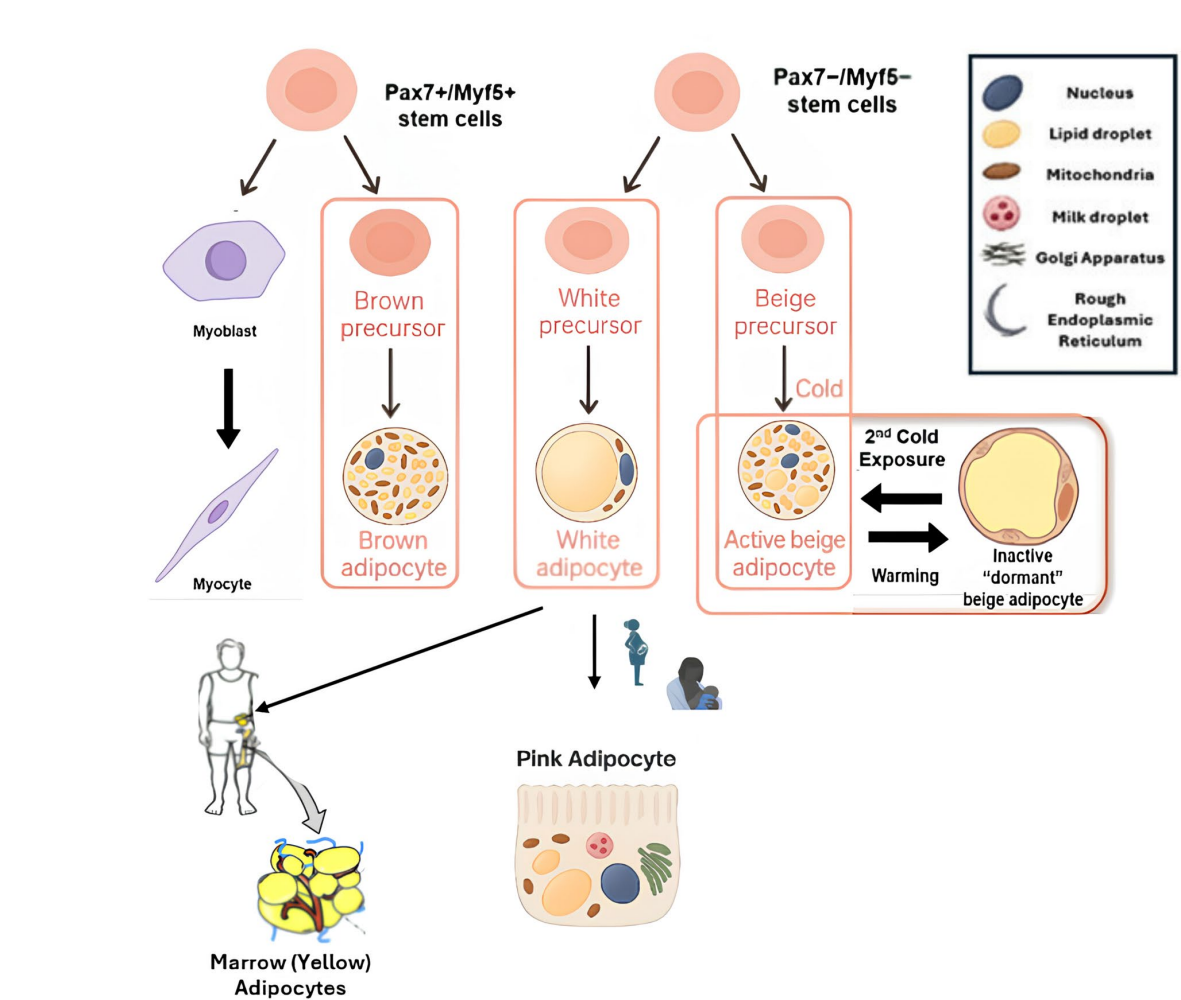
Adipocyte types [18, 19, 21]. This figure illustrates the lineage origins and functional transitions of brown, white, beige, and pink adipocytes. Brown adipocytes derive from Pax7⁺/Myf5⁺ stem cells, whereas white and beige adipocytes originate from Pax7⁻/Myf5⁻ precursors. Cold exposure drives beige precursor cells to form multilocular, thermogenic beige adipocytes, which revert to a dormant unilocular state upon warming but can be reactivated by subsequent cold. During pregnancy and lactation, white adipocytes transdifferentiate into milk-producing pink adipocytes, demonstrating reversible adipocyte plasticity. Bone marrow yellow adipose tissue originates from a lineage closely related to white adipocytes and represents a distinct adipose depot with unique regulatory roles in skeletal metabolism and hematopoiesis. Abbreviations: Myf5: Myogenic Factor 5, Pax7: Paired Box Gene 7

Adipose tissue is a highly dynamic organ that plays a crucial role in maintaining systemic homeostasis [14]. It is composed primarily of adipocytes, along with a small number of other cell types [6]. Adipocytes, which are derived from a common mesenchymal stem cell precursor shared with muscle and bone, differentiate into distinct cell lineages under the control of transcription factors. There are different types of adipocytes, including white, brown, beige, and pink [15]. Adipose tissues are classified according to the predominance of these adipocyte types [13]. These can be broadly categorized into three main types: WAT, BAT, and beige (brite) adipose tissue [9]. Although WAT and BAT share certain structural similarities, such as heterogeneous cellular compositions, extensive vascularization, and innervation, they differ in their metabolic regulation, resulting in distinct impacts on human health [16]. The characteristics of these three adipocyte types are presented in Table 1 [17, 18]. In addition to WAT, BAT, and beige adipose tissue, pink adipose tissue has been identified in recent years. First described in 2014 in female mice during pregnancy and lactation, these cells originate from white adipocytes and acquire the capacity to form milk-secreting alveoli. Morphologically, they resemble epithelial cells and are rich in mitochondria, peroxisomes, and rough endoplasmic reticulum. Pink adipose tissue has been reported to undergo dynamic and reversible transdifferentiation during pregnancy, lactation, and the postlactation period. Although its existence in humans has not been confirmed, this transition is thought to provide important insights into breast biology and tumorigenesis [19]. Another distinct adipose depot within this broad spectrum of adipose tissues is bone marrow adipose tissue (BM-AT, yellow). BM-AT constitutes more than 10% of total fat mass in lean and healthy individuals [20]. Technological advances in the quantitative imaging of BM-AT in both mice and humans have revealed unique features that highlight the physiological specificity of this depot. However, knowledge regarding the phenotype of primary bone marrow adipocytes (BM-Ad) remains limited, largely due to difficulties in isolating sufficient numbers of these cells from rodents and technical challenges associated with accessing human BM-AT. Numerous studies have demonstrated that BM-AT increases under various pathophysiological conditions, including aging, osteoporosis, and obesity. These findings suggest that BM-Ad are not merely passive space-filling cells, but instead play important roles in metabolic and cellular processes. Unlike other WAT depots, caloric restriction leads to an increase in BM-Ad number and cell size in both animal models and individuals with anorexia nervosa, whereas a reduction in BM fat is observed only during severe nutrient deprivation and in the late stages of anorexia nervosa [21]. All types of adipose tissue are illustrated together in Fig. 1 [18, 19, 21].

## White Adipocytes

WAT is widely distributed throughout the body and is located mainly in visceral and subcutaneous regions [13]. It is composed primarily of white adipocytes [10]. These cells contain a single, large lipid droplet that occupies most of the cell volume. They have few mitochondria, and the nucleus is pushed to the periphery of the cell [13], giving them their characteristic white appearance [15]. White adipocytes are particularly effective at energy storage and release [1, 10, 15], storing energy in the form of triglycerides [22]. White adipocytes are healthy at normal sizes [23]; however, in response to excessive energy intake and lipid accumulation [6], adipose tissue undergoes a dynamic remodeling process [1]. Adipose tissue expansion occurs through hypertrophy (enlargement of adipocytes), hyperplasia (increase in adipocyte number), or both mechanisms [10], and this condition is closely associated with obesity [9]. While hyperplasia results in less inflammation [15], hypertrophy can lead to tissue hypoxia and subsequent interstitial fibrosis, ultimately impairing the metabolic function of adipose tissue [24]. WAT also functions as an endocrine organ that secretes various adipokines, hormones, growth factors, enzymes, and cytokines. These endocrine signals play critical roles in determining the metabolic regulatory function of WAT. In contrast, the characteristics of WAT observed in obese individuals include increased secretion of leptin and resistin, decreased adiponectin secretion, reduced insulin sensitivity, elevated basal lipolysis, low vascular density, increased macrophage infiltration, a proinflammatory macrophage profile, and predominantly visceral distribution [15]. Such disturbances in metabolic homeostasis [16], particularly dysfunction caused by excessive visceral fat accumulation, trigger a wide range of health problems, including metabolic syndrome, type 2 diabetes, cardiovascular diseases, and cancer [14]. The anatomical division of WAT into subcutaneous and visceral depots reflects not only positional differences but also substantial distinctions in developmental origin, histological architecture, and metabolic function. Notably, visceral adiposity is linked to increased mortality and metabolic risk, whereas subcutaneous WAT is generally considered more protective. These depots also differ in their thermogenic capacity, as the browning potential of visceral and subcutaneous WAT is markedly distinct [25]. Histologically, subcutaneous WAT exhibits greater heterogeneity, comprising both small multilocular and mature unilocular adipocytes, whereas visceral fat appears more homogeneous and consists predominantly of large unilocular adipocytes. In rodents, subcutaneous depots have been reported to develop earlier than visceral depots. Collectively, these differences underscore the divergent contributions of subcutaneous and visceral WAT to metabolic health and highlight their distinct physiological impacts on metabolic outcomes [26].

## Beige Adipocytes

In recent years, significant progress has been made in the field of brown fat biology, and one of these advances has been the identification of two types of thermogenic adipocytes in humans with distinct developmental origins: classical brown adipocytes and beige adipocytes, which can be induced within WAT [22]. Beige adipocytes are a newly defined type of adipocyte located within WAT but phenotypically resemble brown adipocytes [13]. These cells are developmentally distinct from “classical” brown adipocytes, which arise from myogenic factor 5 (Myf5)-positive myoblastic cells [11]. Under conditions of positive energy balance, adipose tissue remodeling is driven primarily by the proliferation of progenitor cells and their differentiation first into preadipocytes and subsequently into hyperplastic adipocytes [14]. The vascular system provides platelet-derived growth factor receptor alpha–positive (PDGFRα⁺) progenitor cells, creating a microenvironment for adipogenesis; these progenitors possess the capacity to differentiate into both beige and white adipocytes [23]. Beige adipocytes are distinguished from white adipocytes by their multiple small lipid droplets [15]. Initially, they display a white adipocyte-like phenotype, but upon stimulation, they acquire a brown fat-like phenotype, leading to increased thermogenesis. This process, known as browning, occurs predominantly in subcutaneous fat depots [13]. The browning of WAT refers to the appearance of thermogenically active cells within white fat regions that exhibit high mitochondrial density and express UCP1 [10, 15].

Beige adipocytes functionally exhibit characteristics similar to those of classical brown adipocytes [14]. Both types of adipocytes express UCP1, contribute to thermogenesis, and are stimulated by cold exposure and β-adrenergic signaling [9, 27]. The thermogenic activity of brown and beige adipocytes, which generate energy by oxidizing fatty acids and glucose, can effectively reduce adipocyte size [6]. Consequently, brown and beige adipose tissues have been shown to exert protective effects against glucose intolerance and obesity-related metabolic disorders [14]. Therefore, browning, which increases energy expenditure, has emerged as a novel approach for the prevention or attenuation of obesity and other metabolic dysfunctions [24].

**Table 2.**
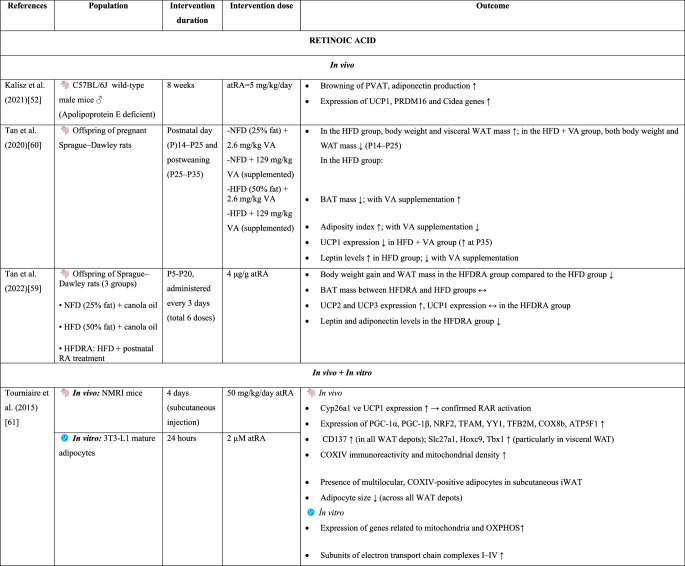

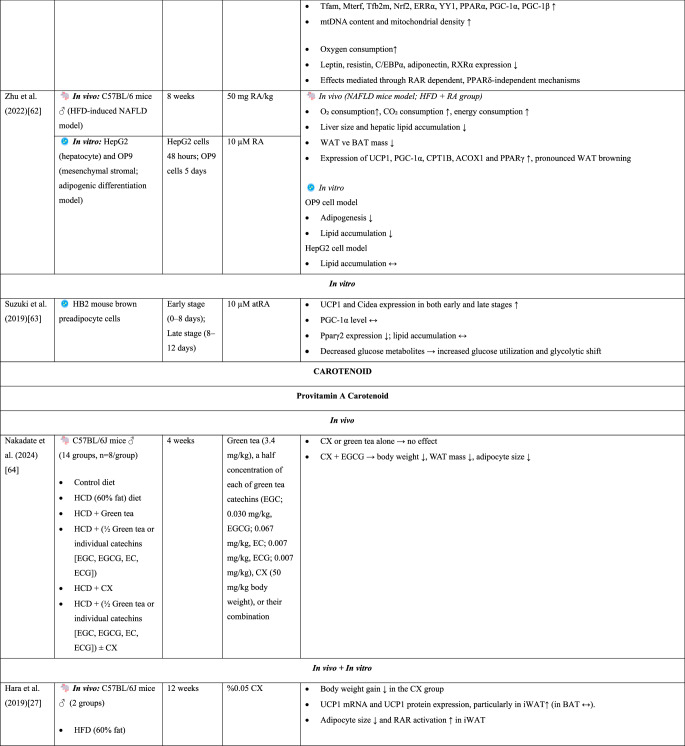

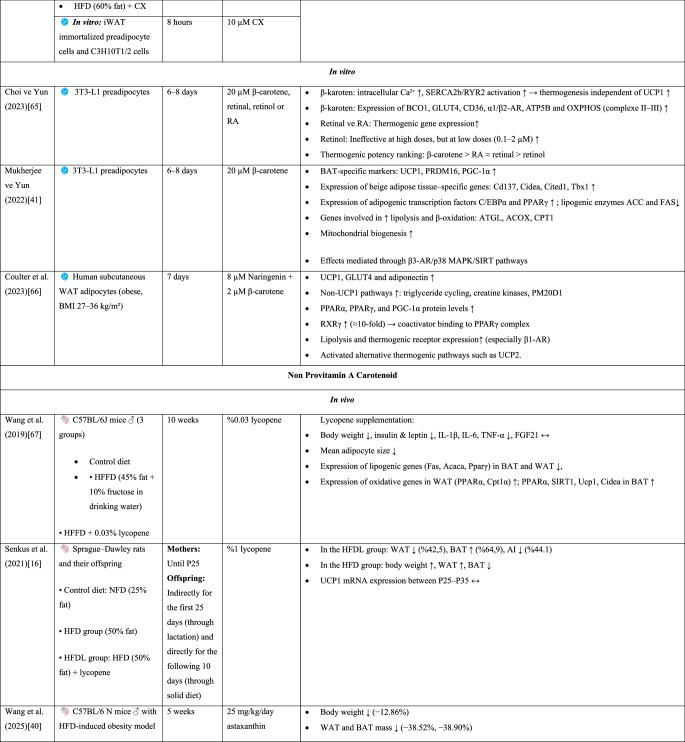

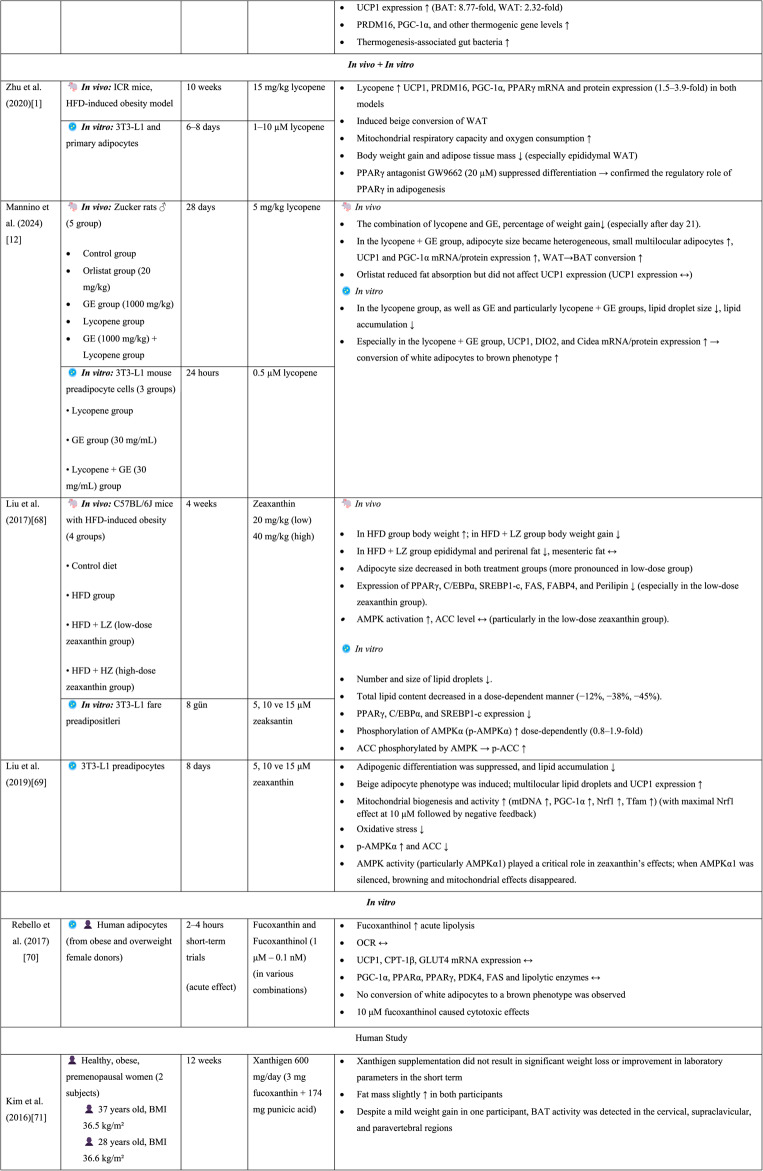
*In vivo* and *in vitro* findings on the effects of vitamin A and its derivatives on brown fat activation and adipose tissue browning

Abbreviations: ACC: Acetyl-CoA Carboxylase, ACOX1: Acyl-CoA Oxidase Type 1, AI: Adiposity Index, AMPK: AMP-Activated Protein Kinase, ATGL: Adipose Triglyceride Lipase, ATP5B: ATP Synthase F1 Subunit Beta, ATP5F1: ATP Synthase F1 Subunit F1, AtRA: All-Trans Retinoic Acid, BAT: Brown Adipose Tissue, BCO1: Beta-Carotene Oxygenase 1, BMI: Body Mass Index, CD137: Cluster of Differentiation 137, CD36: Cluster of Differentiation 36, C/EBPα: CCAAT/Enhancer Binding Protein Alpha, Cidea: Cell Death-Inducing DFFA-Like Effector A, CITED1: CBP/p300-Interacting Transactivator with Glu/Asp-Rich C-Terminal Domain 1, COX8b: Cytochrome c Oxidase Subunit 8B, COXIV: Cytochrome c Oxidase Subunit IV, CPT: Carnitine Palmitoyltransferase, CX: β-Cryptoxanthin, CYP26A1: Cytochrome P450 Family 26 Subfamily A Member 1, DIO2: Type 2 Iodothyronine Deiodinase, EC: Epicatechin, ECG: Epicatechin Gallate, EGC: Epigallocatechin, EGCG: Epigallocatechin Gallate, ERRα: Estrogen-Related Receptor Alpha, FABP4: Fatty Acid-Binding Protein 4, FAS: Fatty Acid Synthase, FGF21: Fibroblast Growth Factor-21, GE: Garcinia Cambogia Extract, GLUT4: Glucose Transporter Type 4, HCD: High-Calorie Diet, HFD: High-Fat Diet, HFDL: High-Fat Diet + Lycopene, HFDRA: High-Fat Diet + Postnatal Retinoic Acid Treatment, HFFD: High-Fat High-Fructose Diet, Hoxc9: Homeobox C9, HZ: High Dose, IL-1β: Interleukin-1 Beta, IL-6: Interleukin-6, iWAT: Inguinal White Adipose Tissue, LZ: Low Dose, MTERF: Mitochondrial Transcription Termination Factor, NFD: Normal-Fat Diet, NRF1: Nuclear Respiratory Factor 1, NRF2: Nuclear Respiratory Factor 2, OCR: Oxygen Consumption Rate, OXPHOS: Oxidative Phosphorylation, P: Postnatal Day, p38 MAPK/SIRT: p38 Mitogen-Activated Protein Kinase/Sirtuin Family, PDK4: Pyruvate Dehydrogenase Kinase 4, PGC-1α: Peroxisome Proliferator-Activated Receptor Gamma Coactivator 1-Alpha, PGC-1β: Peroxisome Proliferator-Activated Receptor Gamma Coactivator 1-Beta, PM20D1: Peptidase M20 Domain Containing 1, PPARδ: Peroxisome Proliferator-Activated Receptor Delta, PPARγ2: Peroxisome Proliferator-Activated Receptor Gamma 2, PRDM16: PR Domain Containing 16, PVAT: Perivascular Adipose Tissue, RA: Retinoic Acid, RAR: Retinoic Acid Receptor, RXR: Retinoic X Receptors, RYR2: Ryanodine Receptor 2, SERCA2b: Sarco/Endoplasmic Reticulum Ca²⁺-ATPase 2b, Slc27a1: Solute Carrier Family 27 Member 1, SREBP1-c: Sterol Regulatory Element-Binding Protein 1c, TBX1: T-Box Transcription Factor 1, TFAM: Mitochondrial Transcription Factor A, TFB2M: Transcription Factor B2 Mitochondrial, TNF-α: Tumor Necrosis Factor Alpha, UCP1: Uncoupling Protein 1, UCP2: Uncoupling Protein 2, UCP3: Uncoupling Protein 3, VA: Vitamin A, WAT: White Adipose Tissue, YY1: Yin Yang 1, α1/β1/β2/β3-AR: Alpha-1/Beta-1/Beta-2/Beta-3 Adrenergic Receptors

## Brown Adipocytes

While WAT constitutes the body’s main energy reserve, BAT is responsible for energy dissipation through energy expenditure and thermogenesis [13]. In humans, BAT develops from Myf5-positive mesenchymal precursor cells that share a common lineage with muscle cells [15]. It is composed primarily of brown adipocytes [10]. These cells are polygonal in shape and, unlike white adipocytes, contain numerous small lipid droplets, a centrally located nucleus, a clear cytoplasm, and abundant mitochondria [1, 13], which give them their characteristic brown coloration [15]. Although BAT is more prominent in rodents, its presence and function have also been demonstrated in humans [14], where it is distributed mainly in the cervical, supraclavicular, paravertebral, mediastinal, and perirenal regions [13]. The amount of BAT in adults is relatively limited; some studies have reported that BAT depots constitute approximately 1.5% of total body weight and 4.3% of total fat mass [28]. The volume and function of BAT vary among human populations; for example, individuals of South Asian descent have been reported to have a lower BAT volume than White Caucasians do [6].

BAT is involved in no shivering thermogenesis [12]. Its thermogenic function is driven primarily by the high oxidative capacity of brown adipocytes, their dense mitochondrial content, and the expression of the characteristic UCP1 protein [10]. These cells initiate heat production by oxidizing glucose and lipids through their abundant oxidative mitochondria [12]. The UCP1 protein, located in the inner mitochondrial membrane, is subsequently activated, allowing protons to leak across the membrane. This proton leak uncouples oxidative phosphorylation, dissipating the electrochemical gradient as heat rather than producing ATP [1]. This mechanism plays a crucial role in preventing hypothermia [6]. In addition, BAT exhibits increased energy expenditure associated with high nutrient utilization, which enhances total body energy expenditure and has the potential to reduce body weight and WAT accumulation [16]. Mice lacking UCP1 exhibit impaired thermogenesis and increased susceptibility to diet-induced obesity [1]. Increasing energy expenditure is one of the most effective ways to prevent excessive adipocyte expansion, and BAT plays a central role in this process. It is estimated that BAT contributes approximately 25–100 kcal of daily energy expenditure [6]. However, the amount of BAT in adults is relatively low, suggesting that its effect on obesity may be limited [9]. For this reason, the rediscovery of BAT has greatly stimulated research interest in this area [22]. The induction of UCP1-expressing beige adipocytes within WAT (browning) and the activation of BAT have gained increasing attention for their ability to improve glucose and lipid profiles and enhance whole-body energy expenditure, thereby contributing to obesity prevention and management [1]. In a study by Dinas et al., a significant negative correlation was found between body mass index (BMI) and BAT activity; individuals with a normal BMI presented greater BAT activity than overweight and obese subjects did [29].

### BAT Activation and Browning

 Many nutritional and signaling cues that activate BAT thermogenesis also promote the beiging of WAT [10]. In humans, BAT activation and browning are stimulated by exposure to cold or the administration of β-adrenergic receptor agonists [11, 12]. The thermogenic function of BAT provides an evolutionary advantage that enables animals to survive under cold conditions [6]. When animals are exposed to cold, temperature sensors located on sensory neurons at the body surface (transient receptor potential channels, TRP) transmit this information to the brain, leading to increased activity of the sympathetic nerves innervating BAT [22]. As a result, BAT becomes activated, increasing both the number of brown adipocytes and the amount of UCP1 produced through the proliferation of interstitial preadipocytes and mature adipocytes. This process enhances energy expenditure and reduces body fat [11, 22]. Prolonged cold exposure also induces the appearance of UCP1-positive adipocytes within WAT, resulting in browning [11]. In a study conducted by Hibi et al., cold exposure (19 °C) led to increased diet-induced thermogenesis and fat utilization in BAT-positive individuals, indicating a physiological role of BAT in energy metabolism [30]. Although this approach is undoubtedly the most physiological and effective way to activate and recruit BAT, it may also increase cardiovascular risk in patients [11, 12].

 Adrenergic stimulation also triggers BAT activation through β3-adrenergic receptors (β3-ARs), initiating a thermogenic process in which transcriptional regulators such as PPARγ, PGC-1α, and PRDM16 increase mitochondrial biogenesis and UCP1 expression [1, 13, 15]. β3-AR agonists can promote this effect by activating signaling pathways such as mitogen-activated protein kinase (MAPK), cyclic adenosine monophosphate (cAMP), and AMPK, thereby increasing the expression of thermogenic genes during browning [31, 32]. Peroxisome proliferator-activated receptors (PPARs) belong to a nuclear receptor family classified as ligand-activated transcription factors, and three subtypes have been identified: PPARα, PPARβ/δ, and PPARγ, which share structural and functional similarities [33]. Human adipose tissue differentiation largely depends on the actions of PPARγ, which plays a central role in adipogenesis and is considered the master regulator of this process. In the absence of PPARγ, adipose tissue development is limited and irregular [15]. Furthermore, PPARγ has been shown to regulate the expression of the adiponectin gene, the adiponectin receptor, and the UCP1 gene while suppressing the expression of inflammatory genes [33]. In a study by Loft et al., PPARγ agonists such as rosiglitazone were shown to promote browning and enhance mitochondrial oxidative capacity in both human and rodent adipocytes. Rosiglitazone increased the expression of thermogenic genes, including UCP1, and this gene program remained active even after treatment cessation [34]. Conversely, Rachid et al. demonstrated that a PPARα agonist enhanced browning and thermogenesis in high-fat diet–fed animals by upregulating UCP1, transmembrane protein 26 (TMEM26), and PRDM16 expression, whereas a PPARβ/δ agonist suppressed UCP1 expression and favored a white adipocyte phenotype. The authors concluded that PPARα activation could be effective in the treatment of metabolic diseases [35]. PPARα also regulates carbohydrate and lipid metabolism by increasing free fatty acid oxidation, improving insulin sensitivity, and promoting energy expenditure. It modulates lipolytic and thermogenic genes in both hepatocytes and brown adipocytes. Specifically, in BAT, PPARα-mediated transcriptional regulation acts in cooperation with PGC-1α: PPARα activates PGC-1α, which in turn enhances UCP1 gene expression and induces PRDM16, establishing a positive feedback loop that reinforces PGC-1α expression [36]. Mechanistically, PRDM16 regulates thermogenic genes by binding to the promoters of UCP1 and PGC-1α and interacting with PGC-1α to support brown adipocyte function [37]. PRDM16 has been identified as a key regulator of brown adipogenesis by directing the differentiation of brown adipocytes from Myf5-positive progenitors shared with myoblasts [31, 37]. While PPARγ alone can induce the conversion of myogenic cells into adipocytes, PRDM16 expression is required for their differentiation into brown adipocytes [37]. PGC-1α has been identified as a binding partner of PPARγ in brown adipocytes [31]. is enriched in BAT and other thermogenic tissues. UCP1 activation is mediated by PGC-1α via irisin (Fibronectin type III domain-containing protein 5, FNDC5), initiating the browning process [38]. Pharmacological approaches used to induce browning include PPARα agonists, adrenergic receptor stimulation, thyroid hormone administration, and irisin induction. Most of these agents act through PGC-1α to promote mitochondrial biogenesis and UCP1 induction [13]. The use of β3-AR agonists can stimulate UCP1 expression and, consequently, the browning process [12]. In a study by Cero et al., treatment of human adipocytes with a β3-AR agonist increased lipolysis, elevated intracellular cAMP levels, and significantly upregulated UCP1 and PGC-1α mRNA expression in differentiated adipocytes. However, no marked increase in the oxygen consumption rate or energy expenditure was observed. These findings suggest that β3-AR activation strongly promotes lipolysis in human white/beige adipocytes, but their thermogenic capacity may be limited [39]. Nevertheless, the clinical use of such agents in obesity treatment is limited by their reduced efficacy and adverse effects [12]. Therefore, identifying alternative, safe, and effective strategies to activate adipose thermogenesis has become an urgent priority [40].

 The use of natural compounds with medicinal properties is considered a strategic approach for treating obesity [41]. Recent studies have revealed that dietary components, pharmaceuticals, and lifestyle conditions may offer new opportunities to intervene in this process [1]. BAT activation and browning are also stimulated by various postprandial humoral factors, such as bile acids and gut hormones, and by certain dietary components that affect the TRP sympathetic nervous system axis [11]. Some of these include dietary compounds such as catechins, capsaicin, resveratrol, curcumin, specific lipid classes, and plant extracts. These bioactive substances can enhance BAT activation through TRP channels and various metabolic pathways, mimicking the effects of cold exposure. One of the nutritional compounds studied in this context is retinoic acid (RA) [11, 13]. In recent years, VA has gained attention for its potential benefits in the prevention of metabolic diseases [42].

### Vitamin A

 VA was discovered in 1915 through the studies of McCollum and Davis, followed by Osborne and Mendel. It is generally used to refer to a group of retinoid compounds possessing the biological activity of all-trans retinol [43]. VA is a fat-soluble vitamin and an essential nutrient, as humans cannot synthesize compounds with VA activity de novo [44]. In human nutrition, VA is obtained from two sources: preformed VA (retinol and retinyl esters) found in animal-derived foods and provitamin A carotenoids (β-carotene, α-carotene, and β-cryptoxanthin) found among plant pigments [45]. To exhibit provitamin A activity, a carotenoid molecule must contain at least one free β-ionone ring and an appropriate number and position of methyl groups along the polyene chain [46]. Theoretically, carotenoids can yield two molecules of VA [42]. Other carotenoids that do not serve as VA precursors (nonprovitamin A carotenoids) include several xanthophylls, such as lycopene, canthaxanthin, lutein, and zeaxanthin, which play significant functional roles in maintaining human health [43].

 The recommended dietary allowances (RDAs) for VA are expressed in retinol activity equivalents (RAEs) to account for the differing biological activities of retinol and provitamin A carotenoids, as all of these compounds are converted to retinol by the body [45]. The RDA for adults over 19 years of age is 900 μg RAE for men and 700 μg RAE for women [47]. Since VA is stored in the body, excessive intake may lead to accumulation and toxicity, a condition known as acute hypervitaminosis A, which typically occurs at extremely high doses, approximately 100 times the RDA [45]. The effects of hypervitaminosis A on the liver, the primary storage site of vitamins, are multifaceted and include hyperplasia and hypertrophy of fat-storing cells [43]. The tolerable upper intake level (UL) for VA is set at 3,000 μg RAE for individuals aged 19 years and older [47]. Nevertheless, VA deficiency remains a major public health problem in developing countries, primarily due to inadequate intake and limited accessibility of animal-derived VA and provitamin A carotenoids. Populations at highest risk include premature infants, children, pregnant and lactating women, and individuals with certain health conditions [45]. In Turkey, data from the 2017 Turkey Nutrition and Health Survey indicate that 26.6% of the population consumes less than the estimated average VA requirement, suggesting that approximately one in four individuals is at risk of VA deficiency [48]. Moreover, recent studies have shown that overweight and obese young adults have significantly lower VA intake than their normal-weight peers [49–51].

**Fig. 2:**
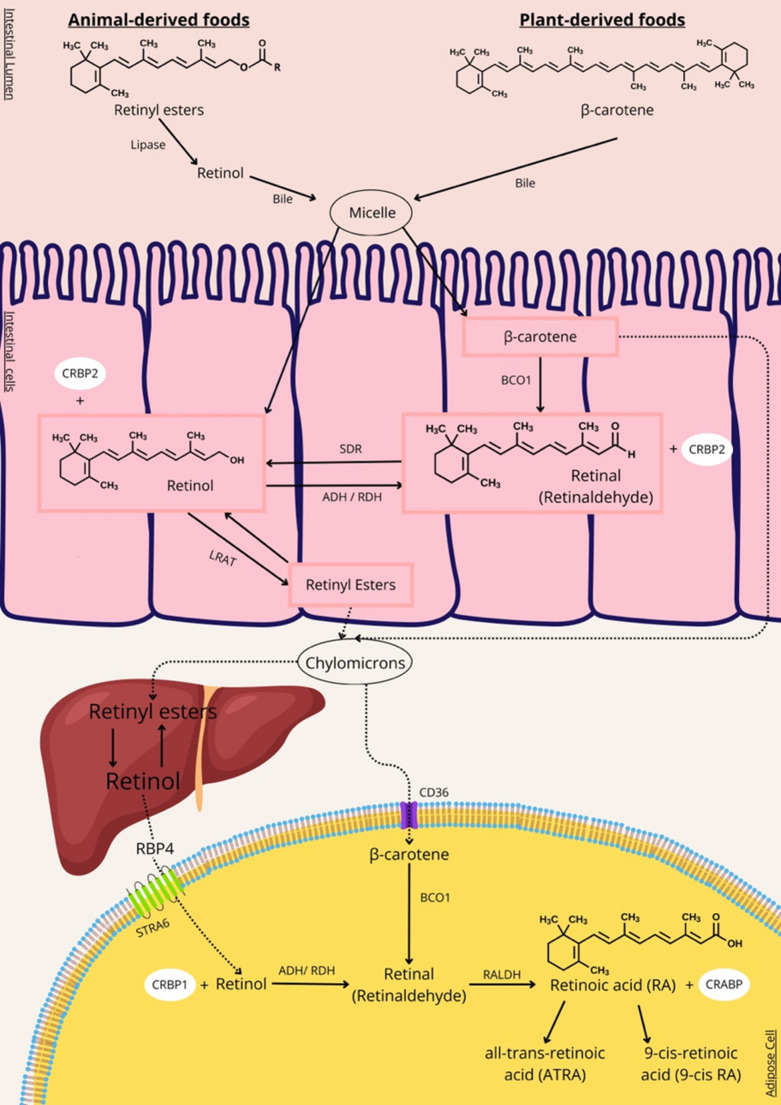
Metabolic conversion of β-carotene and retinoids in adipose tissue [53]. This schematic illustrates the digestion, absorption, transport, and intracellular conversion of vitamin A compounds derived from animal- and plant-based foods, as well as their subsequent metabolism within adipose tissue. (1) Intestinal lumen: Retinyl esters from animal-derived foods are hydrolyzed by luminal lipases to produce free retinol, which subsequently incorporates into bile salt–containing mixed micelles. Similarly, β-carotene and other provitamin A carotenoids from plant-derived foods are solubilized in mixed micelles formed in the presence of bile, allowing their delivery to the intestinal epithelium. (2) Uptake of micelles by enterocytes, re-esterification, and chylomicron assembly: Mixed micelles deliver dietary retinol and β-carotene to the apical membrane of enterocytes, where they are taken up. Inside the enterocyte, β-carotene is cleaved by BCO1 to generate two molecules of retinal (retinaldehyde). Retinol arriving via micelles can be oxidized to retinal by ADH/RDH (RDH1 and RDH10), whereas retinal generated from β-carotene can be reduced back to retinol through SDR family (DHRS3 and DHRS9). CRBP-II binds both retinol and retinal, stabilizing these intermediates within the enterocyte. Subsequently, retinol is esterified to retinyl esters by LRAT. These retinyl esters are incorporated into nascent chylomicrons together with dietary lipids. Chylomicrons are then transported into the lymphatic system and subsequently enter the bloodstream. (4) Hepatic uptake and storage: Chylomicron remnants deliver retinyl esters to the liver. Within the liver, retinyl esters are predominantly stored in hepatic stellate cells, whereas retinol may either be temporarily stored, re-esterified, or mobilized. Retinol is released into the circulation bound to RBP4, allowing its transport to peripheral tissues. (5) Delivery to peripheral tissues: Retinol binds to RBP4 in the liver and is released into circulation as the holo-RBP complex. Circulating holo-RBP is recognized by the STRA6 membrane receptor on adipocytes, enabling the cellular uptake of retinol. (6) Direct β-carotene uptake by adipocytes: Circulating β-carotene can also be taken up by adipocytes via the CD36 transporter. Intracellular β-carotene undergoes BCO1-mediated cleavage and follows the same conversion pathway to retinal and RA. (7) Adipocyte metabolism: Within adipocytes, retinol binds to CRBP1, which stabilizes intracellular retinol and facilitates its channeling toward oxidative metabolism. Retinol is oxidized to retinal by ADH/RDH enzymes and subsequently to RA by RALDH. Both ATRA and 9-cis-RA can be generated. CRABP chaperone RA toward nuclear receptors. Abbreviations: ADH: Alcohol dehydrogenase, ATRA: All-trans-retinoic acid, BCO1: β-carotene oxygenase-1, CD36: Cluster of differentiation 36, CRABP: Cellular retinoic acid–binding proteins, CRBP: Cellular retinol-binding protein, DHRS: DHRS: Dehydrogenase/Reductase, LRAT: Lecithin:retinol acyltransferase, RA: Retinoic acid, RALDH: Retinaldehyde dehydrogenase, RBP: Retinol-binding protein, RDH: Retinol dehydrogenases, SDR: Short-chain dehydrogenase/reductase, STRA6: Stimulated by retinoic acid 6

 Because VA depends on other dietary components, it requires partial digestion before absorption in the small intestine [43]. Provitamin A carotenoids have specific absorption and metabolic pathways and exhibit lower biological activity than do preformed VA forms such as retinol or retinyl esters [46]. While retinyl esters are hydrolyzed by lipase enzymes in the intestinal lumen to form retinol, provitamin A carotenoids are first converted to retinal and subsequently to retinol within enterocytes through the action of the enzyme β-carotene oxygenase 1 (BCO1) [45]. Retinol consists of a β-ionone ring, a polyunsaturated side chain, and a polar end group. Owing to this structure, it has poor water solubility but can easily diffuse across the lipid bilayers of cell membranes [46]. Retinol is further oxidized in the body to produce two major active metabolites of VA: retinal and RA [45]. RA is the active metabolite responsible for most of the biological effects of VA [42]. The two biologically active metabolites of RA (all-trans retinoic acid (atRA) and 9-cis RA) regulate a wide range of physiological functions through two distinct receptors: retinoic acid receptors (RARs), which are activated by both all-trans and 9-cis RA, and retinoid X receptors (RXRs), which are activated exclusively by 9-cis RA [52]. This regulatory mechanism influences the expression of more than 500 target genes involved in cell differentiation and development, reproduction, immune function, and the regulation of lipid and energy metabolism [46]. Figure 2 schematically illustrates the absorption of dietary VA from animal and plant sources and its metabolic conversion within adipose tissue [53].

 VA functions as a key regulator of cellular differentiation processes and plays a crucial role in adipogenesis [54]. During the early stages of adipogenesis, signaling pathways initiated by VA before PPARγ activation contribute to the induction of the differentiation program. In mature adipocytes, the activation of RAR and PPARβ/δ receptors by RA enhances lipid oxidation and energy expenditure, thereby contributing to the reduction in fat accumulation. Experimental studies in mouse models have demonstrated that RA treatment significantly increases UCP1 expression and suppresses the development of obesity [24]. In a study conducted by Correa-Rodríguez et al., VA levels in healthy young adults were found to be inversely associated with obesity-related parameters such as fat mass and BMI [49]. Similarly, Ju et al. reported consistent findings in a Chinese population, showing that VA intake exerted a protective effect against overweight and obesity, particularly among women [50].

### VA and BAT Activation and Browning

**Fig. 3:**
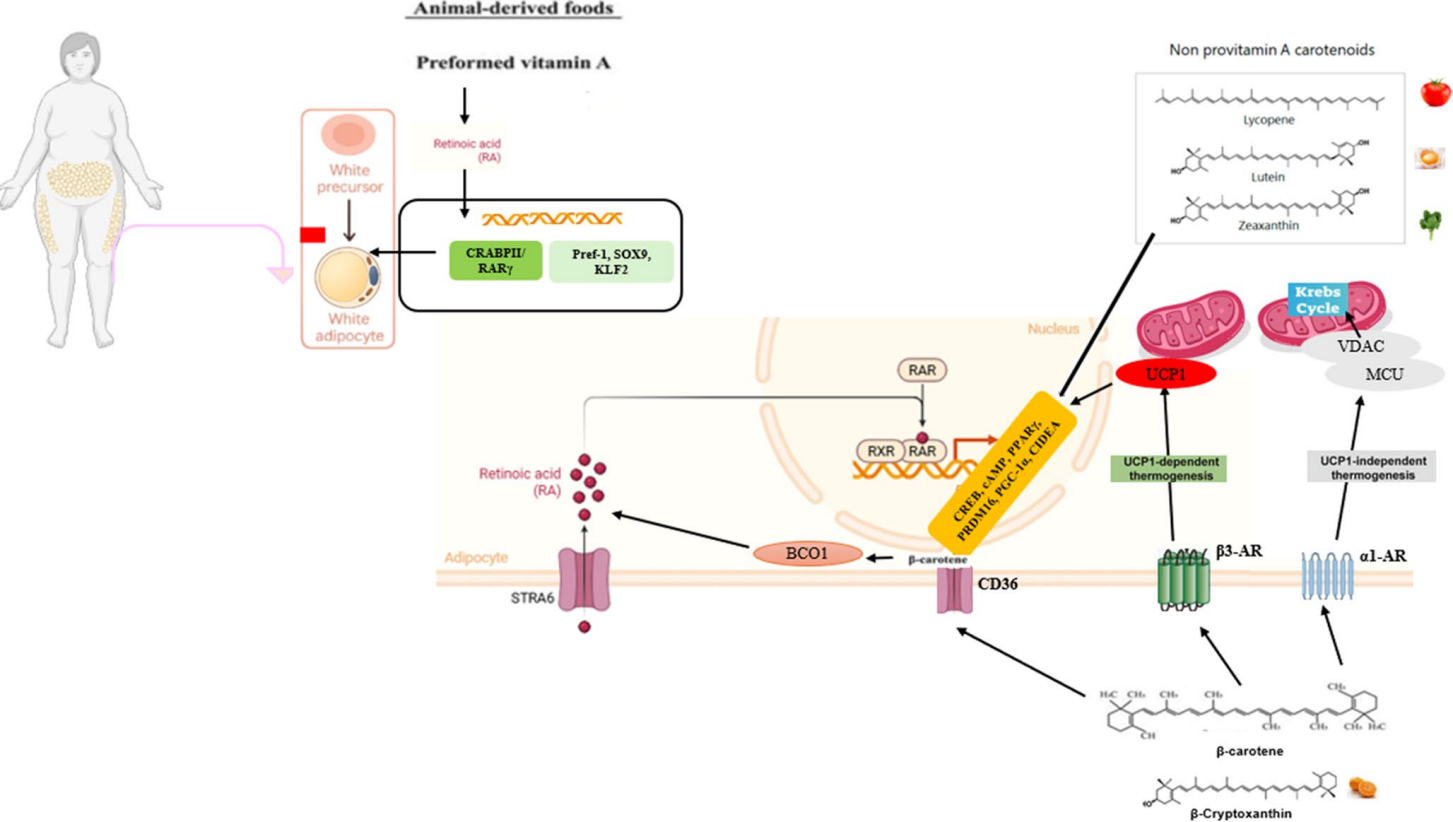
Molecular mechanisms of vitamin A derivatives in adipocyte differentiation and browning. This schematic summarizes the regulatory effects of animal-derived preformed VA (retinol and retinyl esters), plant-derived provitamin (β-carotene and β-cryptoxanthin) and non-provitamin A carotenoids (lycopene, lutein, zeaxanthin) on white adipocyte biology and thermogenesis. (1) Preformed vitamin A pathways: Circulating retinol is taken up into adipocytes via STRA6 and converted into RA. RA is then directed to nuclear receptors (RAR/RXR). Activation of the RA–RAR/RXR pathway increases anti-adipogenic factors such as Pref-1, SOX9, and KLF2, thereby suppressing early stages of adipogenesis. (2) Thermogenic effects of RA: RA induces the transcription of key brown/beige-associated genes such as UCP1, PRDM16, PGC-1α, and Cidea in mature adipocytes. This activation enhances both mitochondrial biogenesis and oxidative capacity, thereby promoting UCP1-dependent thermogenesis. (3) Metabolism of provitamin A carotenoids: Provitamin A carotenoids are taken up into adipocytes via CD36 and are enzymatically cleaved by BCO1 to yield two molecules of retinal. The subsequent conversion of retinal to RA activates retinoid signaling pathways that induce the metabolic programs described above. (4) Thermogenic mechanisms regulated by carotenoids: Provitamin A carotenoids enhance UCP1-dependent thermogenesis by activating the β3-AR, leading to the upregulation of UCP1 and other brown/beige-associated genes such as PRDM16, PGC-1α, and Cidea, similar to the transcriptional actions induced by RA. In addition, provitamin A carotenoids stimulate α1-AR signaling, promoting UCP1-independent thermogenesis through the induction of VDAC–MCU–mediated mitochondrial Ca²⁺ cycling. (5) Non–Provitamin A Carotenoids and Thermogenic Activation: Non–provitamin A carotenoids such as lycopene, lutein, and zeaxanthin exert regulatory effects on adipose tissue independently of conversion to retinoids. In mature adipocytes, they upregulate thermogenic genes (e.g., UCP1, PGC-1α, PRDM16). Abbreviations: BCO1: Beta-carotene oxygenase 1, cAMP: Cyclic adenosine monophosphate, CD36: Cluster of Differentiation 36, Cidea: Cell death–inducing DNA fragmentation factor alpha-like effector A, CREB: cAMP response element–binding protein, CRABPII: Cellular retinoic acid–binding protein II, KLF2: Krüppel-like factor 2, MCU: Mitochondrial calcium uniporter, PGC-1α: Peroxisome proliferator–activated receptor gamma coactivator 1-alpha, PPARγ: Peroxisome proliferator–activated receptor gamma, Pref-1: Preadipocyte factor 1, PRDM16: PR domain–containing 16, RA: Retinoic acid, RAR: Retinoic acid receptor, RXR: Retinoid X receptor, SOX9: SRY-box transcription factor 9, STRA6: Stimulated by Retinoic Acid 6, UCP1: Uncoupling protein 1, VA: Vitamin A, VDAC: Voltage-dependent anion channel, α1-AR: Alpha-1 adrenergic receptor, β3-AR: Beta-3 adrenergic receptor

 Obesity is now recognized not only as an increase in body weight but also as a systemic disease that triggers oxidative stress and inflammation at the cellular level. Multiple factors are involved in this complex process, ranging from leptin resistance to the expression of proinflammatory markers [55]. A detailed understanding of the molecular mechanisms regulating adipogenesis is essential for developing strategies to overcome obesity and its associated pathologies [56]. In recent years, VA has emerged as a particularly noteworthy molecule [57]. Through its ability to suppress adipocyte maturation while promoting thermogenesis and browning, VA exerts significant anti-obesity effects. VA and its derivatives (retinoids) play multifaceted roles in the regulation of adipose tissue metabolism. Figure 3 schematically illustrates the molecular mechanisms through which VA derivatives influence adipocyte differentiation and browning. RA limits the differentiation of preadipocytes into mature adipocytes by suppressing the expression of key transcription factors involved in adipogenesis, including PPARγ and C/EBPα.

 Moreover, through the cellular retinoic acid–binding protein II/retinoic acid receptor gamma (CRABP-II/RARγ) axis, RA has been shown in cell-based models to increase the expression of early anti-adipogenic regulators such as preadipocyte factor 1 (PREF-1), sex-determining region Y-box transcription factor 9 (SOX9), and Kruppel-like factor 2 (KLF2) [56]. In adipose tissue, retinoids also regulate brown fat-related and browning-associated pathways. RA has been reported to increase thermogenic gene expression, particularly UCP1, in both BAT and the conversion of WAT to a beige phenotype [58]. Likewise, postnatal atRA administration in Sprague–Dawley rats (4 µg/g from P5 to P20, six total doses) increased the expression of UCP1, UCP2, and UCP3 in BAT, indicating that vitamin A derivatives converge on mitochondrial thermogenic programming [59]. Additionally, RA promoted WAT browning by stimulating the differentiation of PDGFRα⁺ adipocyte progenitors, a finding demonstrated in vivo in mouse adipose tissue following RA treatment at physiologically active doses [24]. Human data support these observations. Galmés et al. reported that individuals carrying vitamin-A-sensitive genotypes (e.g., UCP1 rs1800592) showed higher adiposity and metabolic impairment under low VA intake, whereas adequate intake ameliorated fat accumulation [7]. Complementary evidence from developmental models shows that maternal VA supplementation in Sprague–Dawley rats, administered at 2.6 mg/kg or 129 mg/kg during lactation and post-weaning, reduced high-fat diet-induced WAT expansion, increased BAT mass, alleviated oxidative stress, and improved lipid and adipokine profiles in offspring, collectively mitigating metabolic disturbances [60]. Various animal and cell culture based studies conducted in this field are summarized in Table 2.

### Effect of Retinoic Acid on Browning

**Fig. 4:**
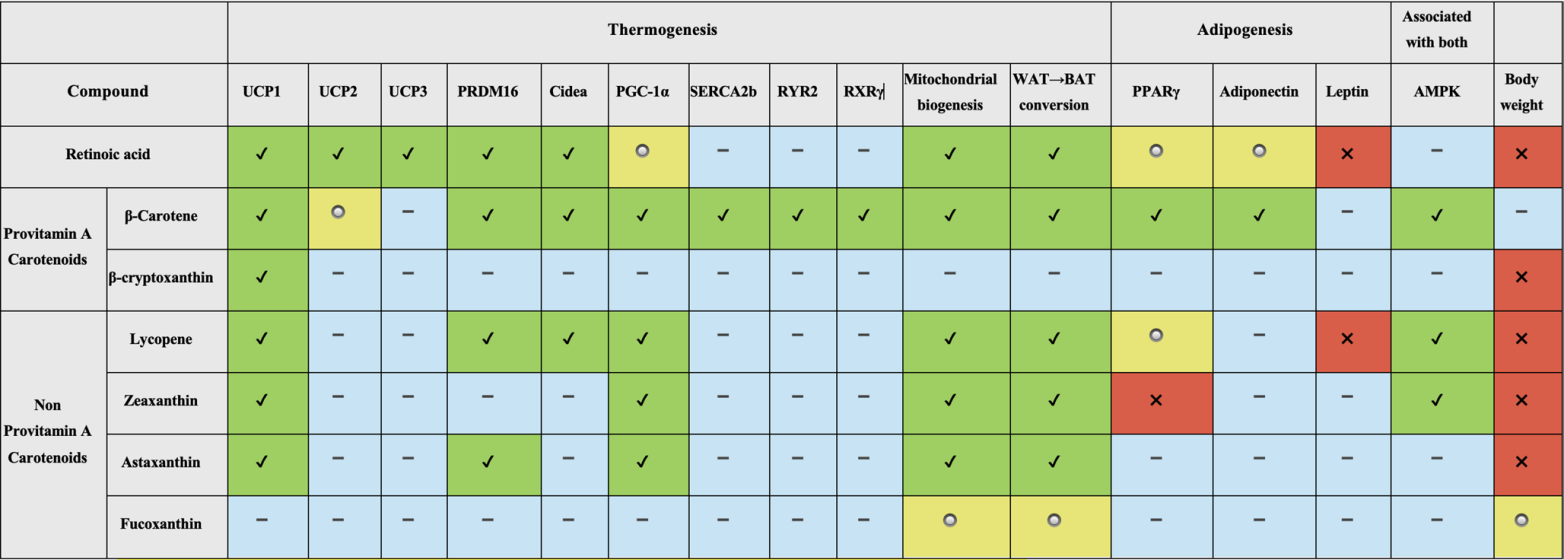
Overview of the molecular effects of retinoids and carotenoids on adipose tissue thermogenesis and adipogenesis ✔: Increase (upregulation/improvement), ▬:No significant change, ✖: Decrease (downregulation/impairment), :Mixed, unclear, or limited data Abbreviations: AMPK: AMP-activated protein kinase, Cidea: Cell death–inducing DNA fragmentation factor alpha-like effector A, PPARγ: Peroxisome proliferator–activated receptor gamma, PGC-1α: Peroxisome proliferator–activated receptor gamma coactivator 1-alpha, PRDM16: PR domain–containing 16, RYR2: Ryanodine receptor 2, RXRγ: Retinoid X receptor gamma, SERCA2b: Sarco/endoplasmic reticulum Ca²⁺-ATPase 2b, UCP: Uncoupling protein

 Retinoids, regulate adipose tissue development and metabolic homeostasis by modulating pathways mediated by nuclear receptors that control adipogenesis, lipid storage and thermogenesis [6]. RA exerts a stage-dependent effect during fat cell development. In early stages, RA acts as an anti-fat cell factor that inhibits fat cell differentiation by suppressing the master transcriptional regulators PPARγ and C/EBPα [72]. Additionally, RA increases energy expenditure by regulating genes involved in lipid metabolism and mitochondrial dynamics. In mature adipocytes, RA activates both RARs and PPARβ/δ, thereby enhancing lipid oxidation and promoting energy utilization rather than storage; at the same time, it induces UCP1, PGC-1α, and other oxidative genes, which strengthen thermogenic activation and mitochondrial function [6, 24]. It promotes the differentiation of stem cells into adipocyte precursors and facilitates the conversion of lipid-storing white adipocytes into thermogenically active brown/beige adipocytes through the regulation of transcription factors such as zinc finger protein 423 (ZFP423) and PRDM16 [6]. At the molecular basis of this transformation, the promoter region of the UCP1 gene contains specific binding sites for the thyroid hormone receptor, PPAR, and RA receptors (RAR and RXR) [52]. The PPAR response elements (PPAR-RE) and RARE located in these regions mediate the transcriptional regulation of UCP1 expression. Through this structure, RA serves as a strong inducer of the UCP1 gene, independent of adrenergic stimulation [16, 73]. PPARs form heterodimers with RXR, bind to the promoter regions of target genes, and play a critical role in adipogenesis by regulating gene expression [15, 66]. RA acts as a natural ligand for RXR and plays an essential role in the functionality of the complexes formed by RXR [66]. RA also serves as an endogenous ligand not only for RXR but also for RAR-type nuclear receptors, thereby playing a critical role in the function of these complexes [42]. Together, these mechanisms contribute to reduced lipid accumulation and, consequently, to weight loss (Fig. 4).

 atRAis the biologically most active form of RA [56]. *In vitro,* Suzuki et al. demonstrated that atRA treatment (10 μM) increased the expression of the UCP1 and cell death-inducing DFFA-like effector A (Cidea) in brown adipocytes, leading to changes in the levels of cellular metabolites related to glucose metabolism, whereas PGC-1α expression remained unaffected. Early atRA treatment (days 0–8) regulated the differentiation of brown preadipocytes, whereas treatment during later stages (days 8–12) was effective in both mature HB2 brown adipocytes and preadipocytes [63]. Across *in vivo *diet-induced and genetic mouse models, atRA consistently enhances thermogenic programming, although with model-specific differences. Krois et al. demonstrated that the absence of retinol dehydrogenase 1 (RDH1), an enzyme required for physiological atRA synthesis (Fig 2), led to increased adiposity without changes in caloric intake, followed by the development of glucose intolerance and insulin resistance. RDH1 deficiency impaired mitochondrial function by reducing UCP1, oxygen consumption, and the membrane potential in BAT. This study highlights the physiological importance of endogenous AtRA signaling [74]. In another study by Tan et al., three groups of offspring were compared: (1) offspring of dams fed a normal-fat diet (NFD) during pregnancy (the offspring received no supplementation), (2) offspring of dams fed a high-fat diet (HFD) during pregnancy (the offspring received no supplementation), and (3) offspring of HFD-fed dams whose offspring received postnatal RA supplementation. Compared with those in group 1, the offspring in group 2 presented significantly lower tissue retinol levels and higher body weights, WAT masses, serum leptin and adiponectin levels, and gene expression of these adipokines. In contrast, in the RA-supplemented offspring (group 3), retinol levels were markedly increased, whereas all other parameters were significantly reduced. In addition, UCP2 and UCP3 gene expression in WAT was greater in the RA-treated offspring than in the control offspring. Collectively, these findings indicate that RA supplementation during lactation improves vitamin A status in neonates exposed to maternal obesity, reduces body weight gain and fat accumulation, and modulates adipose metabolism to prevent obesity. These results suggest that RA functions as a developmental regulator of adipose tissue, shaping long-term thermogenic and endocrine function [59]. Kalisz et al. reported that treatment with atRA (5 mg/kg/day for 8 weeks) induced UCP1 expression in both C57BL/6J and apolipoprotein E-deficient (Apo-E) mice. However, a significant increase in the expression of browning-related genes such as Cidea and PRDM16 was observed only in Apo-E mice, supporting the browning of abdominal perivascular adipose tissue. The authors proposed that this strain-specific response may relate to regulatory differences, including miRNA-133–mediated PRDM16 repression and the distinct modulatory roles of PGC-1α in the two models [52]. Zhu et al., using a non-alcoholic fatty liver disease (NAFLD) model, demonstrated that RA primarily targets WAT rather than hepatocytes. In HFD-fed C57BL/6J mice, RA promoted browning by regulating the expression of thermogenic genes such as UCP1, PPARγ, and PGC-1α in WAT and increased UCP1 levels in BAT, thereby increasing energy expenditure and alleviating disease symptoms. However, the presence of hepatic steatosis and inflammation in NAFLD models may alter adipose responses to RA, limiting generalization of these findings [62]. Tourniaire et al. demonstrated that atRA induced UCP1-expressing beige adipocytes in subcutaneous WAT, whereas in visceral WAT, it increased mitochondrial biogenesis and oxidative phosphorylation capacity without UCP1 expression. These effects were confirmed by both *in vitro* and *in vivo* increases in mitochondrial content, revealing that atRA can increase energy expenditure through both UCP1-dependent and UCP1-independent mechanisms. Although the authors reported no toxicity despite the high-dose atRA administration (50 mg/kg/day) and noted that subcutaneous delivery leads to gradual dilution, this acute and chronic pharmacological dosing regimens still differ markedly from typical dietary VA exposure in humans, limiting the direct translatability of these findings [61]. 

 In addition to the role of atRA in adipocyte biology, retinol-binding protein (RBP) plays a central role in the transport and systemic balance of retinoids [75]. Three types of RBPs are responsible for the intracellular transport of retinol and its metabolites. One of these proteins, RBP7, is involved in lipid and whole-body energy metabolism. Kim et al. reported that RBP7, under the regulation of PPARγ, supports adipogenesis and that its overexpression increases adipocyte differentiation and triglyceride accumulation. During this process, genes related to PPARγ and retinol metabolism are activated, intracellular RA levels are elevated, and the retinoic acid response element (RARE) response is strengthened. These findings indicate that RBP7 regulates adipocyte formation by increasing nuclear RA availability and suggest that its overactivity may contribute to obesity development, making it a potential target for obesity treatment [54]. On the other hand, RBP4 is the only specific carrier of circulating retinol and is highly expressed in the liver and adipose tissue. Park et al. developed transgenic mice expressing RBP4 in BAT and reported that these mice presented increased energy expenditure, reduced body fat, and increased thermogenesis in response to cold. Increased lipolysis and fatty acid oxidation in BAT are associated with adrenergic signaling; moreover, retinol levels are increased in BAT but decreased in the liver. These results indicate that the overexpression of RBP4 in BAT promotes energy expenditure and thermoregulation, suggesting that RBP4 is a potential target for the treatment of metabolic disorders [76]. In the study by Fenzi et al., cold exposure increased circulating retinol and RBP levels; however, in RBP4 knockout mice, WAT browning, thermogenic gene expression, and mitochondrial function were impaired, and body temperature decreased rapidly under cold conditions. In human adipocytes, retinol increases mitochondrial respiration and thermogenic gene expression, and in humans, cold-induced retinol elevation is associated with increased lipid oxidation. These findings indicate that retinol supports thermogenesis and energy expenditure in adipocytes through its conversion to RA via the RAR and RXR pathways [75]. and consistent with this mechanism, the results from in vitro models have demonstrated that RA interacts with RAR-α to activate specific RARE regions within the UCP1 gene, thereby increasing UCP1 mRNA expression in brown adipocytes [16]. Wang et al. reported that RA suppressed adipogenesis through RAR activation via epigenetic mechanisms, and this effect weakened the white adipocyte phenotype while promoting browning and energy expenditure [77]. While these findings demonstrate the effects of RA on adipogenesis and browning, adipokines that mediate this process have also been identified. One of these adipokines, lipocalin-2 (Lcn2), has been reported to be an important adipokine that regulates the thermogenic activation of brown adipose tissue and RA-induced thermogenesis in mice [78]. The promoter region of the Lcn2 gene contains multiple transcription factor-binding sites and nuclear receptor response elements, particularly RARE [73]. Deis et al. demonstrated that Lcn2 deficiency led to mitochondrial dysfunction by reducing UCP1 and PGC-1α expression. The increase in thermogenic genes observed with combined RA and insulin treatment was abolished in the absence of Lcn2; Lcn2 contributed to beige adipocyte activation by increasing UCP1 expression through the p38 MAPK pathway [78]. Guo et al. further showed that Lcn2 is a target gene of RA and that atRA increased the expression of UCP1 and Lcn2 in adipocytes. Lcn2 deficiency attenuated the effects of atRA on obesity, lipid metabolism, and thermogenesis, whereas retinoid transport was disrupted through the stimulation by retinoic acid 6 (STRA6) and RBP4 pathways. These findings revealed that Lcn2 serves as a critical regulator of RA-mediated thermogenesis and retinoid homeostasis [73].

 RA has been shown to regulate not only adipocyte differentiation and thermogenesis but also the vascular structure of adipose tissue. RA promotes angiogenesis in adipose tissue by activating vascular endothelial growth factor (VEGF) signaling, thereby increasing the number of PDGFRα⁺ adipose progenitor cells surrounding blood vessels [6, 24]. Through this mechanism, RA induces browning of WAT. These effects of RA disappear when VEGF receptors are conditionally knocked out, indicating that browning is dependent on VEGF signaling [24]. Wang et al. reported that maternal supplementation with 30 IU/ml VA in mice increased the number of PDGFRα⁺ progenitor cells and vascular density in offspring, which was associated with beige adipogenesis and metabolic health. Pharmacological or genetic inhibition of the RA signaling pathway abolished these effects, confirming the requirement for RA activity. Moreover, the action of RA is mediated via vascular endothelial growth factor receptor 2 (VEGFR2), but this effect is supported not only by angiogenesis but also by independent mechanisms. Genes involved in browning, including PRDM16, are upregulated, adipocyte size is reduced, and offspring are protected against diet-induced obesity [23]. Another study demonstrated that RA promoted vascularization in WAT and beige adipogenesis of PDGFRα⁺ progenitor cells by increasing vascular endothelial growth factor A (VEGFA) expression. Increased vascularization increases the number of differentiable cells, whereas RA directs these cells toward the beige adipocyte lineage, suggesting a critical role in preventing obesity [24]. Together, these findings indicate that RA remodels adipose tissue through both angiogenesis-dependent and angiogenesis-independent mechanisms, although most evidence derives from mouse models with high-dose or developmental interventions, factors that may limit direct translation to human physiology.

### Effect of Carotenoids on Browning

 The mechanisms underlying the browning effects of carotenoids largely involve their conversion into retinoid metabolites and the activation of nuclear receptor–driven thermogenic pathways. Provitamin A carotenoids such as β-carotene and β-cryptoxanthin can be enzymatically converted to retinal and subsequently RA, which activates RAR/RXR-mediated transcriptional programs that induce UCP1, PGC-1α, PRDM16, and other oxidative genes, thereby promoting thermogenesis and mitochondrial biogenesis in adipocytes [16, 27, 42]. Beyond retinoid pathways, β-carotene also activates β3-AR, cAMP, p38 MAPK, and SIRT3/6 signaling, enhancing lipolysis, fatty acid oxidation, and beige adipocyte formation [41, 65]. In addition, combined carotenoid actions, such as the synergistic effects of naringenin and β-carotene, upregulate UCP1 and glucose transporter type 4 (GLUT4) while markedly increasing RXRγ expression and facilitating RXR–PPARγ coactivator interactions, further promoting mitochondrial function and adaptive thermogenesis [66]. Non-provitamin A carotenoids exert complementary mechanisms; for instance, zeaxanthin activates AMPKα1 signaling, inhibits adipogenic transcription factors, reduces lipid synthesis, and stimulates mitochondrial biogenesis, leading to increased UCP1 expression and beige adipocyte differentiation independently of classical retinoid pathways [68, 69]. Lycopene supports browning by suppressing lipogenic genes while enhancing lipolysis, mitochondrial respiration, and the expression of thermogenic regulators such as UCP1, PGC-1α, and PRDM16; simultaneously, it mitigates inflammation and improves metabolic homeostasis in adipose tissue [1, 67].

### Provitamin A carotenoids

**β-carotene** Among carotenoids, one of the best known and most potent compounds is β-carotene [41, 42]. Epidemiological data suggest that β-carotene may have protective effects against metabolic diseases in which adipose tissue plays a central role. Adipose tissue is one of the primary storage sites for β-carotene, and its function is regulated particularly in response to retinal formation through the BCO1 enzyme and its oxidation to RA. Conversely, the unmetabolized form of β-carotene can also affect adipocyte function [79]. Recent studies have suggested that the bioavailability of β-carotene may be limited, as a large portion undergoes metabolic conversion, and its distribution among tissues plays a significant role [80]. In vivo, Coronel et al. demonstrated that increasing adipose BCO1 levels through gene therapy enhanced RA production from stored β-carotene and led to reductions in adipose mass and adipocyte size [81]. Consistent with these findings, several cell-based studies have clarified the direct actions of β-carotene on adipocytes. In 3T3-L1 preadipocytes, 20 µM β-carotene administered for 6–8 days promoted browning by upregulating UCP1, PRDM16, PGC-1α, and Cidea, while suppressing adipogenic genes and enhancing lipolytic and fatty acid oxidation pathways; these effects were mediated through p38 MAPK–Sirtuin 3/Sirtuin 6 (SIRT3/SIRT6) axis [41]. Similarly, in the study by Choi and Yun, 20 µM β-carotene (retinal, retinol or RA included as comparators) given to 3T3-L1 preadipocytes for 6–8 days increased intracellular Ca²⁺, SERCA2b/RYR2 signaling, and mitochondrial OXPHOS activity, thereby enhancing thermogenesis through both UCP1-dependent and UCP1-independent mechanisms [65]. Complementary evidence from human models was provided by Coulter et al., who treated primary subcutaneous WAT adipocytes derived from obese individuals with 8 µM naringenin plus 2 µM β-carotene for 7 days, leading to increases in UCP1, GLUT4 and adiponectin, activation of non-UCP1 thermogenic pathways, and a nearly tenfold rise in RXRγ, which enhanced coactivator interaction with PPARγ; the combination also improved lipolysis, mitochondrial function, and insulin sensitivity [66].

**β-Cryptoxanthin**, a provitamin A carotenoid with high bioavailability [45], has been shown to have not only a role as a retinol precursor but also direct effects on metabolic functions. Some findings even suggest that the bioavailability of β-cryptoxanthin may be greater than that of β-carotene [82] *In vitro* tests have demonstrated that β-cryptoxanthin increases UCP1 expression in white adipocytes through RAR activation [27]. In the study by Nakadate et al., coadministration of epigallocatechin gallate (EGCG, a green tea catechin) and β-cryptoxanthin for four weeks produced a synergistic effect, leading to significant reductions in body weight and both the mass and size of WAT. It also improved blood lipid profiles and liver functions, indicating systemic beneficial effects on normal physiological functions. The authors state that, due to the synergistic interaction between the compounds, the amount of EGCG required for the anti-obesity effect is reduced to a level that can be easily consumed in daily life. However, the β-cryptoxanthin dose used in the study (50 mg/kg body weight) corresponds to a pharmacological level rather than a nutritionally achievable intake in humans. Therefore, although the study demonstrates a synergistic anti-obesity effect, only the EGCG dose reflects physiologically attainable intake, and the β-cryptoxanthin data are not directly translatable to human nutrition [64]. In studies using C57BL/6J mice receiving 0.05% β-cryptoxanthin for 12 weeks under a high-fat diet, as well as in iWAT preadipocytes and C3H10T1/2 cells treated with 10 µM β-cryptoxanthin for 8 hours, increases in UCP1 expression, reductions in adipocyte size, and enhanced RAR activation were observed. These findings suggest that β-cryptoxanthin may engage RAR-dependent mechanisms, independent of β3-AR signaling, and has the potential to promote thermogenic responses and limit fat accumulation [27].

### Non-provitamin A carotenoids

 Carotenoids such as lycopene, lutein, and zeaxanthin found in foods are not converted into VA [45]; however, they play other important physiological roles in the body, such as antioxidant activity.

**Lycopene**, a red-colored carotenoid, is most abundantly found in tomatoes and tomato-based products [43]. Previous studies have reported that lycopene and its metabolites can stimulate RAR activation in both *in vitro* and animal models, suggesting potential effects on BAT development and activation [16]. In a study by Wang et al., lycopene supplementation at 0.03% for 10 weeks in C57BL/6J mice fed a high-fat, high-fructose diet prevented body weight gain. Lycopene suppressed lipogenic genes while increasing the expression of genes related to lipolysis, thermogenesis, and mitochondrial function (UCPs, PGC-1α, and PRDM16), thereby supporting browning in WAT. It also improved insulin resistance by suppressing inflammation in WAT, regulating leptin and GLUT gene expression, and reducing intestinal permeability [67]. Similarly, Zhu et al. reported that lycopene treatment, administered at 15 mg/kg for 10 weeks in ICR mice and at 1–10 µM for 6–8 days in 3T3-L1 and primary adipocytes, promoted browning in WAT and activation of BAT by upregulating PPARγ, UCP1, PGC-1α and PRDM16 expression. Lycopene treatment reduced body weight, fat mass, and fasting glucose while improving glucose tolerance, insulin sensitivity, the lipid profile, and mitochondrial respiration. It was concluded that lycopene has potential against obesity by regulating energy metabolism [1]. Senkus et al. reported greater body weights and WAT masses in the offspring of mothers fed a HFD, while mothers received a diet containing 1% lycopene until P25 and the offspring were exposed indirectly for 25 days through lactation and directly for an additional 10 days. However, lycopene supplementation during lactation suppressed WAT accumulation, increased BAT development, and reduced serum glucose and lipid peroxides. No significant difference was found in UCP1 expression or other parameters. These results suggest that while lycopene increases BAT mass, it does not necessarily increase its functional activity [16]. In another study, Mannino et al. reported that administering 5 mg/kg lycopene and/or 1000 mg/kg Garcinia cambogia extract to Zucker rats for 28 days, as well as treating 3T3-L1 preadipocytes with 0.5 µM lycopene for 24 hours, increased UCP1, Cidea, and type II iodothyronine deiodinase (DIO2) gene expression and promoted browning and lipolysis. This treatment reduced lipid droplet size while increasing their number and significantly decreased both body weight gain and food intake in rats. Cidea was reported to play a critical role alongside UCP1 and PGC-1α in thermogenesis and adipose tissue loss [12].

**Zeaxanthin** is one of the major naturally occurring xanthophyll carotenoids and is abundant in various foods, such as egg yolk, corn, sea buckthorn, and goji berries. Since the human body cannot synthesize zeaxanthin, its intake relies entirely on dietary sources [69]. In a 2017 study, Liu et al. demonstrated that zeaxanthin administered to C57BL/6J mice for 4 weeks at low (20 mg/kg) or high (40 mg/kg) doses, and applied to 3T3-L1 preadipocytes at 5, 10 or 15 µM for 8 days, activates the AMPK pathway, suppresses the expression of transcription factors and lipid-synthesizing enzymes associated with adipogenesis, inhibits preadipocyte differentiation and lipogenesis, and consequently reduces intracellular lipid accumulation, adipocyte size, epididymal fat mass, and overall body weight. These findings highlight the anti-obesity potential of zeaxanthin [68]. A 2019 study revealed that, in adipocytes treated with 5, 10, or 15 µM zeaxanthin for 8 days, with zeaxanthin, the expression of UCP1 and brown/beige fat markers increased, mitochondrial biogenesis was promoted, and oxidative damage caused by lipid peroxidation was reduced. These effects of zeaxanthin occurred through improvements in the mitochondrial membrane potential and the scavenging of reactive oxygen species and superoxides, and were mediated by AMPKα1 activation, indicating that zeaxanthin exerts anti-obesity effects by promoting beige adipogenesis [69].

**Astaxanthin** is a dietary carotenoid known as a “super antioxidant,” and various studies have confirmed its anti-aging effects and potential health benefits in alleviating obesity. In the study conducted by Wang et al., astaxanthin administered to C57BL/6N mice with HFD-induced obesity at 25 mg/kg/day for 5 weeks was shown to attenuate obesity symptoms. These effects were associated with increased UCP1-mediated thermogenic activation in BAT and browning of inguinal WAT. Astaxanthin administration resulted in a reduction in body weight and fat accumulation, accompanied by a 38.90% decrease in BAT mass and a 38.52% decrease in WAT mass, respectively [40].

**Fucoxanthin**, a carotenoid derived from edible marine algae, has been shown to increase UCP1 expression in the BAT of mice genetically predisposed to obesity and type 2 diabetes, thereby partially alleviating obesity symptoms [13]. Unlike previous animal studies, Rebello et al. found that treating human adipocytes from obese and overweight female donors with fucoxanthin or its metabolite fucoxanthinol at concentrations ranging from 1 µM to 0.1 nM for 2 to 4 hours produced no significant changes in oxygen consumption rate or in the expression of browning and lipid metabolism related genes such as UCP1, PGC-1α, PPARα, PPARγ and lipolytic enzymes. However, compared with the control, fucoxanthinol present in human plasma acutely stimulated lipolysis. The authors concluded that, at physiological doses, fucoxanthin and its metabolite do not promote the conversion of white adipocytes into a beige phenotype [70]. In a case report by Kim et al., two healthy obese premenopausal women were administered 600 mg xanthigen daily (containing 3 mg fucoxanthin and 174 mg punicic acid) for 3 months. The study included no diet or exercise interventions. Although no significant weight loss or laboratory changes were observed, and one participant even experienced slight body weight gain, BAT activity was detected in the cervical, supraclavicular, and paravertebral regions. This finding suggested that xanthigen may stimulate BAT formation [71].

### Is There Truly an Optimal Vitamin Dose for Browning? Can It Be Defined?

 In humans, no specific VA dose that can induce the browning of WAT has yet been identified. Current evidence is largely derived from animal and cell culture studies (Table 2). In human studies, exposure to cold has been shown to increase circulating retinol and RBP levels, which are associated with increased expression of thermogenic genes [75]. In addition to experimental findings, several human and epidemiologic studies have demonstrated that vitamin A status is associated with various metabolic traits. Elevated circulating levels of retinol and its transport protein RBP4 have been linked to obesity, insulin resistance, type 2 diabetes, dyslipidemia, and increased cardiometabolic risk [83]. Furthermore, genetic variants affecting vitamin A metabolism have been shown to modulate metabolic phenotypes. Polymorphisms in the *BCO1* gene reduce the conversion efficiency of β-carotene to retinol, whereas *PNPLA3* variants alter hepatic retinoid storage, thereby increasing susceptibility to metabolic and liver diseases [84]. These findings indicate that vitamin A intake, circulating retinoid biomarkers, and genetic background collectively contribute to shaping metabolic risk profiles in humans. Nutritional authorities have defined safe daily intake limits: 700 µg RAE/day for adult women, 900 µg RAE/day for adult men, and a UL of 3000 µg RAE/day (approximately 10,000 IU) [45, 46].

 Different doses of carotenoids have been tested in human studies [45]. The Expert Group on Vitamins and Minerals has set the safe upper limit for β-carotene at 7 mg/day based on the lowest observed adverse effect level identified in the Alpha-Tocopherol, Beta-Carotene Cancer Prevention (ATBC) study conducted in Finland, where daily β-carotene supplementation of 20 mg was associated with an increased risk of lung cancer among male smokers. The Norwegian Scientific Committee for Food Safety has adopted a more conservative approach, recommending a provisional upper limit of 4 mg/day [46]. Lycopene was used at doses up to 75 mg/day, and lutein was used at doses up to 20 mg/day. The absence of significant adverse effects in published human clinical trials and animal studies involving lutein and lycopene provides strong evidence supporting their safe use in dietary supplements [85]. The typical dietary intake of β-cryptoxanthin ranges from 1–3 mg/day [86]. However, none of these doses have been tested for their effects on UCP1 expression or adipose tissue browning in humans; most studies have focused instead on cancer prevention, cardiovascular health, and eye health. Furthermore, while the European Food Safety Authority (EFSA) has established a safe upper intake level for retinol, it has not set an upper limit for provitamin A carotenoids such as β-carotene because of insufficient data [46]. In this context, it is important to consider that retinoid biology extends beyond metabolic regulation. Retinoid nuclear receptor signaling, activated by the VA metabolite retinoic acid, has been studied for more than a century and exhibits context-dependent, bidirectional effects. Although retinoid agonists provide clear therapeutic benefits in selected clinical settings such as rare skeletal disorders, acute promyelocytic leukemia, and certain dermatologic diseases, pharmacologic activation of this pathway may also lead to adverse outcomes, including acute metabolic disturbances and, under specific tumor microenvironmental conditions, tumor-promoting effects. This duality highlights the need for caution when interpreting the potential metabolic benefits of VA induced adipose tissue browning [87].

 On the basis of current evidence, determining an optimal VA dose to promote browning in humans is not yet feasible. The primary reason is that retinoid metabolism varies significantly across species, and human VA metabolism is highly influenced by environmental, genetic, and hormonal factors [88]. Moreover, interindividual differences in RBP4 levels and hepatic VA stores, which affect VA bioavailability, make establishing a standardized dosage challenging [89].

 To determine the optimal dosage, future dose–response–based, randomized controlled human trials are needed. These studies should comprehensively evaluate not only the effects of VA intake on browning markers such as UCP1, PRDM16, and PGC-1α but also liver function, plasma retinol dynamics, and metabolic safety profiles. Until such data are available, high-dose VA supplementation for browning purposes is not recommended. Instead, maintaining a balanced intake within physiological limits is considered the safest approach [90, 91].

## Conclusion

 Obesity is a complex metabolic disease whose prevalence has rapidly increased in recent years, becoming a major global public health issue. Although various treatment strategies have been developed, recent approaches targeting the activation of BAT and the browning of WAT have gained significant attention. However, the activation of these mechanisms through pharmacological agents or cold exposure is not always feasible or sustainable. Therefore, growing research interest has focused on dietary components capable of naturally modulating these metabolic pathways.

 Among such compounds, VA and its active metabolite atRA have emerged as particularly promising. Evidence in the literature suggests that atRA and certain carotenoid derivatives can increase energy expenditure by stimulating thermogenesis, primarily via UCP1. Provitamin A carotenoids (e.g., β-carotene and β-cryptoxanthin) are known to have lower toxicity than retinol derivatives and are converted to VA only as needed by the body. This property highlights carotenoids as safer potential metabolic modulators. In this context, these compounds may offer potential clinical applications, such as their incorporation into dietary strategies, functional foods, or adjunctive therapeutic approaches aimed at improving metabolic health.

 Nonetheless, most current evidence is still based on animal and *in vitro* studies. Therefore, well-designed clinical trials are needed to clarify the effects of these compounds on adipose tissue browning in humans, to determine optimal dosage ranges, and to establish long-term safety profiles. Additionally, it is important to consider the risk of hypervitaminosis A, interindividual variability in carotenoid turnover, and metabolic disorders before recommending clinical use. Future studies should also focus on personalized nutrition approaches, long-term metabolic monitoring, and the development of targeted strategies to translate these mechanistic findings into clinically applicable interventions.

## Key References

- Ghesmati Z, Rashid M, Fayezi S, Gieseler F, Alizadeh E, Darabi M. An update on the secretory functions of brown, white, and beige adipose tissue: towards therapeutic applications. Rev Endocr Metab Disord. 2024;25(2):279–308. http://doi.org/10.1007/s11154-023–09850-0
  - ○ Comprehensive review summarizing endocrine and paracrine functions of thermogenic adipose tissue, framing how retinoid-induced browning can influence systemic metabolism.
- Wang B, Du M. Increasing adipocyte number and reducing adipocyte size: the role of retinoids in adipose tissue development and metabolism. Crit Rev Food Sci Nutr. 2024;64(29):10608–25. http://doi.org/10.1080/10408398.2023.2227258
  - ○ Comprehensive review describing how retinoids regulate adipocyte proliferation and differentiation through RAR/RXR and PPARγ signaling. It provides the mechanistic foundation for understanding VA’s modulation of adipose metabolism discussed in this review.
- Coronel J, Yu J, Pilli N, Kane MA, Amengual J. The conversion of β-carotene to vitamin A in adipocytes drives the anti-obesogenic effects of β-carotene in mice. Mol Metab. 2022;66:101640. http://doi.org/10.1016/j.molmet.2022.101640
  - ○ Demonstrated that intracellular conversion of β-carotene to VA mediates its anti-obesogenic and thermogenic actions in adipocytes, linking carotenoid metabolism directly to VA-dependent browning mechanisms.

### Future Perspectives

 The multifaceted effects of VA and carotenoids on adipose tissue differentiation, energy metabolism, and thermogenic mechanisms suggest the possibility of an alternative biological pathway that goes beyond classical strategies for combating obesity. These bioactive compounds contribute to restoring metabolic homeostasis through mechanisms that reduce lipid storage, increase mitochondrial activity, and stimulate thermogenesis, thereby increasing energy expenditure.

 Future research should focus not only on descriptive findings but also on advanced clinical studies that elucidate mechanisms of action and dose-response relationships. These studies should aim to determine dose ranges that can safely and effectively stimulate thermogenic activation. Furthermore, the combined evaluation of molecular, metabolic, and imaging-based markers will provide a holistic understanding of the cellular and systemic responses to retinoid and carotenoid interventions. The observation that VA or carotenoid treatments administered to maternal mice produce metabolic effects in their offspring indicates that these compounds may influence metabolic outcomes through fetal and early-life developmental programming. This suggests that VA and its derivatives may function not only as acute metabolic regulators but also as modulators of long-term developmental pathways that shape intergenerational metabolic health. Furthermore, the use of functional compounds such as carotenoids in combination or synergistic treatment methods may provide more potent metabolic responses compared to single-agent strategies. The combined use of these bioactives, particularly those targeting different pathways (e.g., AMPK activation, reduction of oxidative stress, increased mitochondrial biogenesis), may contribute to a more balanced regulation of energy homeostasis.

 Consequently, translating preliminary clinical data into human studies requires multidisciplinary collaboration among the fields of nutritional science, molecular biology, and endocrinology. This will enable the potential of VA and carotenoids as next-generation, epigenetically based metabolic modulators in the fight against obesity to be unearthed.

### Strengths and Limitations

 This literature review is one of the most recent studies to comprehensively evaluate the effects of VA and carotenoids on adipose tissue biology, particularly the browning of white adipose tissue. The examination of numerous studies has enabled a broad perspective on the available evidence, both in terms of diversity and depth. One of the strengths of the study is the comparative analysis of both retinoids and carotenoid derivatives, as well as the integrated analysis of cellular, animal, and limited human data. Furthermore, the combined discussion of multiple biological processes, such as energy metabolism, mitochondrial biogenesis, oxidative stress, and epigenetic regulation, has enabled the topic to be addressed in its biochemical and physiological integrity.

 However, the existing literature is largely based *in vitro* and animal models, limiting the direct generalization of the findings to human physiology. Differences in dose, duration, and model used across studies reduce the comparability of the results. In addition, due to the interindividual variability of VA metabolism and the fact that nutrigenetic and epigenetic factors have not yet been sufficiently elucidated, it is difficult to determine the optimal dose and efficacy level.

## Data Availability

No datasets were generated or analysed during the current study.

## Abbreviations

AMPK: AMP-Activated Protein Kinase
Apo-E: Apolipoprotein E-Deficient
ATBC: Alpha-Tocopherol, Beta-Carotene Cancer Prevention
AtRA: All-Trans Retinoic Acid
BAT: Brown Adipose Tissue
BCO1: Beta-Carotene Oxygenase 1
BM-Ad: Bone Marrow Adipocytes
BM-AT: Bone Marrow Adipose Tissue
BMI: Body Mass Index
C/EBPα: CCAAT/Enhancer Binding Protein Alpha
cAMP: Cyclic Adenosine Monophosphate
Cidea: Cell Death-Inducing DFFA-Like Effector A
CRABP-II: Cellular Retinoic Acid-Binding Protein II
DIO2: Type II Iodothyronine Deiodinase
EFSA: European Food Safety Authority
EGCG: Epigallocatechin Gallate
FNDC5: Fibronectin type III domain-containing protein 5
GLUT4: Glucose Transporter Type 4
HFD: High Fat Diet
KLF2: Kruppel-Like Factor 2
Lcn2: Lipocalin-2
MAPK: Mitogen-Activated Protein Kinase
Myf5: Myogenic Factor 5
NAFLD: Non-Alcoholic Fatty Liver Disease
NFD: Normal Fat Diet
p38 MAPK: p38 Mitogen-Activated Protein Kinase
PDGFRα⁺: Platelet-Derived Growth Factor Receptor Alpha–positive
PGC-1α: Peroxisome Proliferator-Activated Receptor Gamma Coactivator 1-Alpha
PPAR-RE: Peroxisome Proliferator Response Element
PPARs: Peroxisome Proliferator-Activated Receptors
PPARα: Peroxisome Proliferator-Activated Receptor Alfa
PPARγ: Peroxisome Proliferator-Activated Receptor Gama
PRDM16: PR domain containing 16
PREF-1: Preadipocyte Factor 1
RA: Retinoic Acid
RAEs: Retinol Activity Equivalents
RAR: Retinoic Acid Receptor
RARE: Retinoic Acid Response Element
RARγ: Retinoic Acid Receptor Gamma
RBP: Retinol-Binding Protein
RDA: Recommended Dietary Allowance
RDH1: Retinol Dehydrogenase 1
RXR: Retinoic-X-Receptor
SIRT3: Sirtuin 3
SIRT6: Sirtuin 6
SOX9: Sex-Determining Region Y-Box Transcription Factor 9
SREBP1-c: Sterol Regulatory Element-Binding Protein 1c
STRA6: Stimulated by Retinoic Acid 6
TMEM26: Transmembrane Protein 26
TRP: Transient Receptor Potential
UCP1: Uncoupling Protein 1
UL: Tolerable Upper Intake Level
VA: Vitamin A
VEGF: Vascular Endothelial Growth Factor
VEGFA: Vascular Endothelial Growth Factor A
VEGFR2: Vascular Endothelial Growth Factor Receptor 2
WAT: White Adipose Tissue
ZFP423: Zinc Finger Protein 423
β3-AR: Beta 3-Adrenergic Receptor
